# Non-catalytic UBL2 domain directs deubiquitinase USP11 toward K48-linked polyubiquitin chains

**DOI:** 10.1016/j.jbc.2025.110924

**Published:** 2025-11-07

**Authors:** Sin-Rong Lee, Han-Hsiun Chen, Ruey-Hwa Chen, Kuen-Phon Wu

**Affiliations:** 1 Institute of Biological Chemistry, Academia Sinica, Taipei, Taiwan; 2 Institute of Biochemical Sciences, College of Life Science, National Taiwan University, Taipei, Taiwan

**Keywords:** USP1, UBL2, deubiquitinase, polyubiquitin chain, K48-linkage, DiffDock

## Abstract

Ubiquitin-specific proteases (USPs), comprising the largest deubiquitinase family, are generally thought to have poor discrimination of ubiquitin (Ub) linkage types, but a number of USPs show preference toward certain linkages. USP11, a USP-family member implicated in cancer and neurodegeneration, carries an atypical catalytic domain which is split into two segments through the insertion of a UBL2 domain and an intrinsically disordered region (IDR). In addition, the chain-type selectivity of USP11 remains unclear based on the conflicting data from *in vitro* and *in vivo* studies. Here, we identify an important role of the UBL2-IDR in altering the ability of USP11 to cleave K29, K33, and K48 chains, with K48 chain showing the most significant effect. Using *in vitro* studies with Ub-tetramer and ubiquitinated proteins as well as cell-based analyses, we demonstrate that UBL2 domain endows USP11 with a selectivity towards the K48-linked Ub chains. Importantly, this function of UBL2 is not observed in its paralogs USP4 and USP15, which display broad activities towards most chain types. By leveraging AI-based virtual screening, we have identified selective USP11 inhibitors, including the FDA-approved drugs Fenoldopam and Olanzapine and their analogs, which act through a unique chemical scaffold and display significant efficacy both *in vitro* and in cells. Our findings not only uncover a previously unrecognized mechanism of linkage selectivity within the USP family but also provide a robust platform for the rational design of USP11-targeted therapeutics, underscoring the critical role of non-catalytic domains in deubiquitinase regulation and offering promising avenues for therapeutic intervention.

Ubiquitin (Ub), a small 76-amino acid protein, mediates post-translational modifications through conjugation to the target proteins. Ubiquitination can occur as monoubiquitination or polyubiquitination, and the latter exhibits diverse linkages involving eight different Ub chain types: M1, K6, K11, K27, K29, K33, K48, and K63 (1). These topologically different polymers give rise to diverse cellular outcomes, and thus are referred to as “the Ub code” (1, 2).

In contrast to ubiquitination carried out by an E1-E2-E3 enzymatic cascade, the modification can be reversed through a group of enzymes called deubiquitinases (DUBs). DUBs are cysteine proteases or metalloproteases responsible for cleaving Ub molecules from the target proteins, thereby altering their stability, activity, subcellular localization, and signaling (3). DUBs play a key role in many cellular processes and diseases, including DNA repair, transcriptional regulation, membrane transport, cancer, and neurodegeneration and inhibition of DUBs is therefore an attractive strategy in disease therapy (4). Accordingly, small-molecular inhibitors for USP1 and USP30 are currently tested in clinical trials against cancer and renal diseases, respectively (5, 6).

The ubiquitin-specific peptidase (USP) family, the largest DUB family, comprises over 50 members in humans and is characterized by a conserved catalytic domain and variable regulatory regions (3). Different from several other DUB families, such as ovarian tumor-related protease (OTU), Jab1/MPN/Mov34 (JAMM), MINDY and ZUFSP, which display high Ub-linkage specificity, the USP family is generally considered to have poor discrimination of linkages (1, 7, 8, 9, 10). However, several USP family proteases exhibit linkage preferences, such as USP30 (K6), USP37 (K48), USP53/54 (K63), and CYLD (M1 and K63) (11, 12, 13, 14). Thus, the linkage selectivity of USP family members and its underlying mechanism remain incompletely understood.

USP11 is implicated in diverse cellular processes and diseases (15, 16, 17, 18, 19, 20). Studies involving USP11 inhibition or knockdown have revealed its roles in protein stability, transcriptional regulation, cell migration, and chemoresistance (15, 21, 22). For example, USP11 promotes tumorigenesis in prostate cancer by deubiquitinating androgen receptor and c-Myc, as well as removing repressive histone marks (H2A-K119Ub) from their gene promoters (15). Furthermore, USP11 depletion inhibits gastric cancer cell growth and migration *via* the RhoA pathway and has also been implicated in colorectal, breast, ovarian, and liver cancers (20). USP11 also fosters chemoresistance by stabilizing BIP in ovarian cancer (17) and VCP in colorectal cancer (20). Moreover, USP11 interacts with and deubiquitinates tau, leading to tau accumulation and tauopathy (23).

The linkage preference of USP11 has not been well understood. *In vitro* assays indicated that both full-length USP11 and its catalytic core preferentially cleave K6, K33, and K63-linked di-Ub, with little or no activity towards K48-linked di-Ub (24). However, *in vivo* studies demonstrated its activity mainly toward the K48-linked ubiquitinated substrates (17, 21, 25, 26, 27, 28, 29), albeit with the discovery of a few K63-linked ubiquitinated substrates (18, 30). This discrepancy suggests that additional regulatory mechanisms may modulate the chain-type selectivity. In addition, USP11 is composed of an N-terminal DUSP domain, two Ub-fold-like (UBL) domains, a distinctive catalytic domain split into two segments (D1 and D2), and an intrinsically disordered region (IDR) (Fig. 1*A*) (20, 24). Notably, the two segments of the catalytic domain (D1 and D2) are separated by approximately 300 residues through the insertion of a UBL2 and a long IDR. This domain architecture, while uncommon among USP-type DUBs, is also observed in USP4 and USP15, which similarly feature a UBL-IDR insert between the two catalytic domain segments (31). However, the precise role of the UBL2-IDR insert remains an open question with both mechanistic and therapeutic importance.

**Figure 1:**
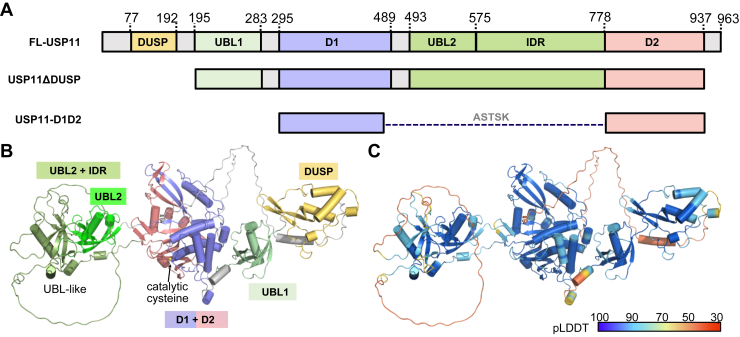
**Domain architecture of USP11 and design of USP11 variants.***A*, full-length USP11 consists of multiple structured domains and one intrinsically disordered region. The catalytic domain is split into D1 and D2, with a UBL2-IDR inserted in between. Two USP11 variants were designed: USP11ΔDUSP, representing the catalytically active form, and USP11-D1D2, representing the catalytic core-only conformation. Notably, D1 and D2 are connected by five residues (ASTSK) (PDB ID: 8OYP (32)). *B*, AlphaFold3-predicted 3D structure of USP11, with structural domains color-coded as in *panel A*. The catalytic core, formed by D1 and D2 segments, resembles the typical USP-type catalytic domain. The UBL2-IDR insert contains a UBL2 domain, a previously uncharacterized UBL-like domain, and a disordered region. *C*, the AlphaFold pLDDT confidence scores for USP11 suggest high confidence in the structured domains; however, the domain–domain interactions may require further experimental validation and refinement.

In this study, we set out to resolve the molecular basis of USP11’s chain specificity and demonstrate that the UBL2, inserted between the split catalytic domain, acts as a key determinant in guiding USP11 for the efficient recognition and processing of K48-linked polyubiquitin chains. In contrast, USP4 and USP15 do not require their UBL2 domains for K48 chain cleavage, highlighting a unique mechanism in USP11. Furthermore, we apply structure-based virtual screening using AI-powered docking to identify selective USP11 inhibitors, including FDA-approved drugs and related analogs. Our findings illuminate the regulatory complexity of USP11, reveal new avenues for DUB-targeted drug development, and provide mechanistic insight with broad relevance to the understanding and pharmacological targeting of deubiquitinases.

## Results

### Distinct deubiquitination mechanism of USP11 beyond the catalytic core

While the full-length structure of USP11 remains undetermined experimentally, AlphaFold predictions suggest that the N-terminal DUSP domain interacts with the UBL1 domain, with the D1 and D2 domains assembling to form the catalytic core (Fig. 1, *B* and *C*). The UBL2-IDR, however, does not appear to directly adjoin the catalytic core. Prior X-ray crystallography of a minimal catalytic domain of USP11 (USP11-D1D2, PDB ID: 8OYP) (32), fused with a crystallization tag, revealed that the D1-D2 domains form a typical USP-fold catalytic core, consistent with the AlphaFold-predicted full-length structure.

**Figure 2:**
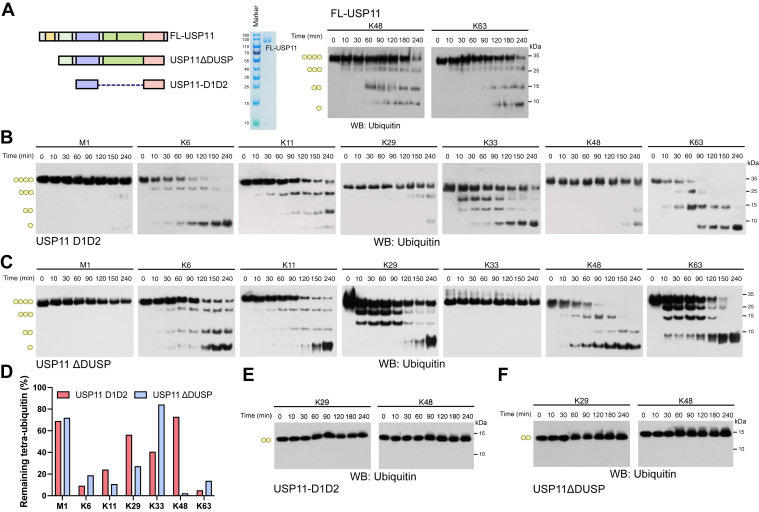
**Deubiquitination and Ub chain selectivity of USP11-D1D2 and USP11ΔDUSP.***A*, FL-USP11 expressed and purified from HEK293T cells was employed to cleave K48 or K63-linkaged Ub_4_ chains, resulting in reduced amounts of Ub_4_ and increased short Ub chains. *B, C*, seven types of Ub_4_ chains were incubated with USP11-D1D2 (*B*) or USP11ΔDUSP (*C*), revealing distinct substrate selectivity and cleavage efficiency. *D*, the amounts of residual Ub_4_ chains after 4-h digestion were evaluated from immunoblots in (*B*) and (*C*) and plotted. (*E*, *F*) USP11-D1D2 (*panel E*) and USP11ΔDUSP (*panel F*) failed to cleave the K29 or K48-linked Ub_2_ chain.

To assess the chain type selectivity of USP11 and the influence of non-catalytic regions on USP11 activity, we generated full-length USP11 (FL-USP11) and two USP11 truncation variants: USP11-D1D2, comprising only the core catalytic region (D1 and D2), and USP11ΔDUSP, which additionally includes the UBL2-IDR region and UBL1 (Figs. 1*A* and S1*A*). While the two variants were purified from *Escherichia coli*, the FL-USP11 was derived from HEK293T cells, due to the difficulty in obtaining large proteins from *E coli*. We first tested the effect of FL-USP11 on K48- and K63-linked tetra-Ub (Ub_4_) chains. We reasoned that Ub_4_ would confer a physiologically more relevant condition than the previously used di-Ub (Ub_2_) chains (24), as Ub_4_ represents the average Ub chain length observed *in vivo* (33). Surprisingly, FL-USP11 cleaved both K48- and K63-linked Ub_4_ chains (Fig. 2*A*), even though a previous study reported the inability of FL-USP11 to act on K48-linked Ub_2_ (24). Given the low yield of FL-USP11 and the irrelevance of the DUSP domain to USP11 catalytic activity (24), we focused on USP11ΔDUSP and USP11-D1D2 for the following studies. To thoroughly characterize the chain type selectivity, a pool of Ub_4_ chains, excluding K27-Ub_4_ due to its commercial unavailability, was employed. Concordant with the previous findings, USP11-D1D2 efficiently cleaved K6, K33, and K63-linked Ub_4_ chains (Fig. 2*B*). Specifically, after a 4-h deubiquitination reaction, the three Ub_4_ chains were >90% cleaved to generate monoubiquitin, whereas M1, K29, and K48-Ub_4_ chains were minimally processed by USP11-D1D2. Notably, K11-Ub_4_ also served as a substrate for USP11-D1D2, as evidenced by the generation of Ub_3_ and Ub_2_ following incubation. These findings reveal a discrepancy in chain type selectivity between FL-USP11 and USP11-D1D2.

Next, the cleavage selectivity of USP11ΔDUSP was examined utilizing Ub_4_ chains (Fig. 2*C*). Consistent with the findings for USP11-D1D2, M1-linked Ub_4_ remained undigested, whereas K6 and K63-linked Ub_4_ exhibited efficient cleavage. However, in marked contrast to USP11-D1D2, USP11ΔDUSP demonstrated minimal cleavage of K33-Ub_4_ chains, even following extended incubation periods. Furthermore, K29 and K48-linkages displayed significantly increased cleavage by USP11ΔDUSP (Fig. 2, *B*–*D*). The reactivity of USP11ΔDUSP on K29 and K48-Ub_4_ chains had not been previously reported but is consistent with the reactivity of FL-USP11 on K48-Ub_4_. Together, these findings suggest that the inserted UBL2-IDR between D1 and D2 domains is crucial for substrate recognition. However, consistent with a previous report (24), when short K29-Ub_2_ and K48-Ub_2_ chains were reacted with USP11-D1D2 (Fig. 2*E*) and USP11ΔDUSP (Fig. 2*F*), no cleavages were observed within 4 h. Our data suggest that the deubiquitination activity of USP11 towards K29 and K48-linked chains is ignited by the longer Ub chains.

**Figure 3:**
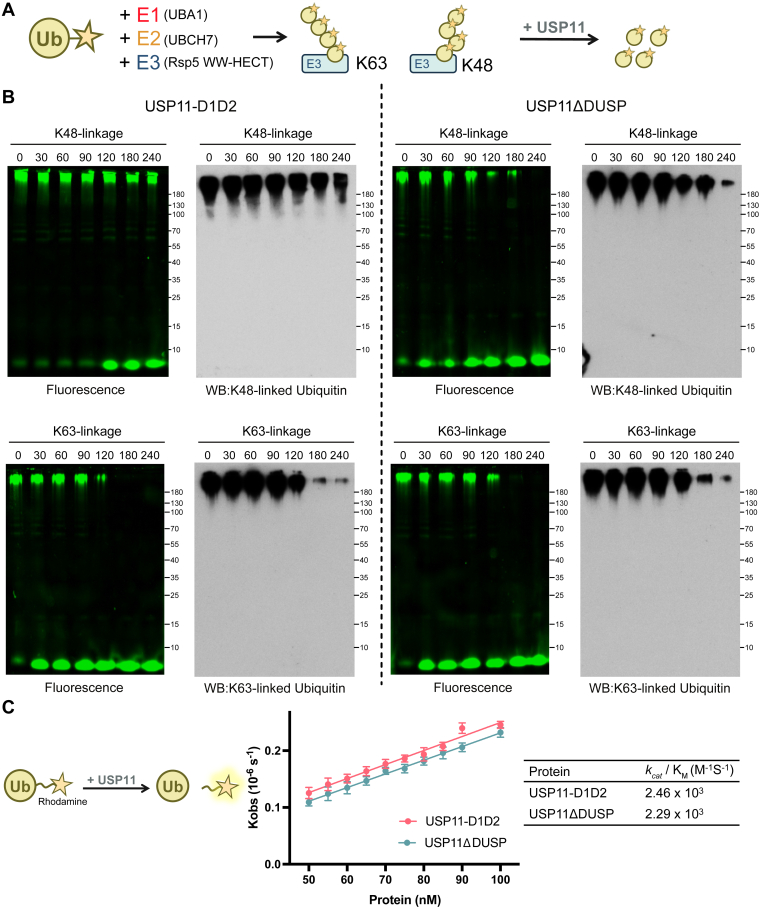
**Selectivity of USP11-D1D2 and USP11ΔDUSP toward K48 and K63 chain-modified substrates and their catalytic efficiency.***A*, polyubiquitinated substrates were generated using UBA1, UBCH7, wild-type or mutant (Q808M-E809L) Rsp5 E3 ligase, together with fluorescein-labeled Ub. The wild-type Rsp5 produced K63-linked polyubiquitin chains, while the mutant form generated K48-linked chains. *B*, USP11-D1D2 and USP11ΔDUSP displayed distinct cleavage patterns toward these substrates. The D1D2 variant was weakly active toward K48-linked chains, whereas USP11ΔDUSP efficiently cleaved both K48- and K63-linked chains, consistent with their activity against Ub_4_ substrates. Chain-specific antibodies were used to confirm the composition and deubiquitination of the K48 and K63-linked substrates. *C*, Ub-Rho was used to evaluate catalytic activity. Initial cleavage rates were measured and plotted against USP11 concentration to determine apparent *k*_*cat*_/K_M_ values.

To further demonstrate the K48 chain selectivity of USP11ΔDUSP on a ubiquitinated protein, we utilized the autoubiquitinated Rsp5 E3 ligase as substrate (Fig. 3*A*). Rsp5, a HECT-type E3 ligase, primarily forms K63-linked Ub chains; however, a C-terminal mutation can switch its specificity towards K48-linked Ub chains (34). We thus leveraged this property to prepare K48- and K63-polyubiquitinated Rsp5 for deubiquitination assays, thus avoiding substrate-specific effects by using different substrates. Consistent with the findings with Ub_4_, the USP11-D1D2 showed weak cleavage of K48-linked Ub chains conjugated to Rsp5. Conversely, USP11ΔDUSP efficiently digested K48-linked Ub chains on Rsp5. Regarding the K63-linked Ub chains conjugated to Rsp5, both USP11-D1D2 and USP11ΔDUSP showed cleavage activity, with the latter displaying a comparatively weaker reactivity (Fig. 3*B*). These observations further support the impact of the UBL2-IDR region on the selectivity of USP11 toward K48-linked Ub chains.

To rule out the possibility that the differential efficacies for USP11ΔDUSP and USP11-D1D2 to cleave K48-linked chains is due to a difference in their overall catalytic activity, rather than chain type selectivity, we utilized ubiquitin-Rhodamine (Ub-Rho) as a substrate (35). Ub-Rho serves as a convenient probe to characterize the enzymatic turnover efficiency of DUBs (36). Once the Rhodamine group is cleaved off, it emits a fluorescent signal at 535 nm, allowing for a real-time measurement of the cleavage activity. Given its monomeric nature, cleavage should not be influenced by chain type selectivity. With this substrate, the apparent *k*_*cat*_/K_M_ values for USP11-D1D2 and USP11ΔDUSP were 2.46 × 10^3^ and 2.29 × 10^3^ M^−1^s^−1^, respectively, revealing comparable catalytic activities (Fig. 3*C*). Thus, the non-catalytic regions affect USP11 chain type selectivity, without significantly altering the overall catalytic efficiency on a monomeric substrate.

### UBL2 uniquely governs the USP11 activity towards K48-linked Ub chains

To elucidate the individual contributions of UBL1 domain and UBL2 domain on Ub chain selectivity, we engineered and purified two additional USP11 variants: USP11-D1D2-IDR, encompassing the D1, D2, and IDR domains, and USP11-USPonly, incorporating the D1, D2, IDR, and UBL2 domains (Fig. S1, *A* and *B*). These variants underwent deubiquitination assays utilizing the K48 and K63-polyubiquitinated Rsp5 as substrates. While the two variants showed no discernible difference in the digestion of K63-linked Ub chains, USP11-USPonly, which does not contain UBL1, exhibited significant cleavage activity towards K48-linked Ub chains on Rsp5, mirroring the effect of USP11ΔDUSP. Conversely, USP11-D1D2-IDR, which lacks both UBL1 and UBL2, showed a minimal cleavage of K48-linked Ub chains conjugated to Rsp5, reminiscent of the effect of USP11-D1D2 (Fig. S1*C*). These findings provide strong evidence that the UBL2, not UBL1, plays a determining role in USP11-mediated K48-linkage recognition and cleavage.

**Figure 4:**
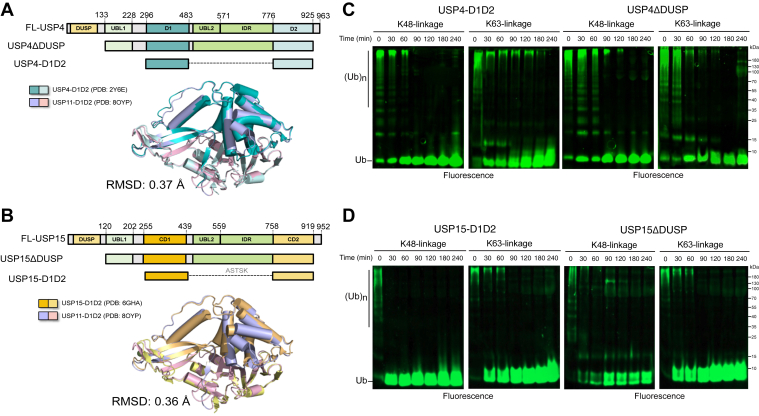
**Ub chain selectivity of USP4 and USP15 is not affected by the UBL2-IDR insert.***A, B*, the domain architectures of USP4 and USP15 follow the same pattern as USP11 (shown in Fig. 1*A*). ΔDUSP and D1D2 variants were also constructed to assess whether the UBL2 insert affects chain selectivity. The D1D2-assembled structures of USP4, USP11, and USP15 are highly similar, with RMSD values less than 0.4 Å. *C*, *D*, both USP4-D1D2 and USP4ΔDUSP cleaved polyubiquitinated Rsp5 substrates with nearly identical patterns, indicating that the UBL2-IDR does not influence chain selectivity in USP4. Similar findings were obtained with USP15-D1D2 and USP15ΔDUSP.

Human USP11, USP4, and USP15 diverge from other USP-family DUBs, forming a distinct subgroup characterized by a conserved domain architecture, including an N-terminal DUSP domain followed by a UBL1-D1-UBL2-IDR-D2 domain arrangement (Fig. 4, *A* and *B*). The catalytic cores of USP4 and USP15 exhibit significant structural similarity to USP11-D1D2, as evidenced by X-ray crystallography, with the root mean square deviation lower than 0.4 Å (Fig. 4, *A* and *B*). Prior studies have elucidated the Ub chain specificity of USP4 and USP15, demonstrating activity towards both K48 and K63-linked chains (31, 37, 38, 39). To assess the linkage selectivity of the catalytic core or DUSP-removed variants, Ub_4_ chains were employed as substrates. In contrast to USP11, USP4-D1D2 and USP4ΔDUSP exhibited comparable cleavage patterns. They preferentially targeted K6, K11, K48, and K63-linked Ub_4_ chains and displayed reduced activity towards K29 and K33-chains and the weakest activity for M1-chains (Fig. S2, A, B). With respect to USP15, USP15-D1D2 and USP15ΔDUSP showed comparable and broad cleavage activity across most Ub_4_ chains, except for M1-Ub_4_, for which USP15-D1D2 exhibited higher reactivity (Fig. S2, *C* and *D*). Collectively, USP4 and USP15 exhibit less stringent linkage preference compared with USP11 and their UBL2-IDR regions play little or no role in determining the chain type preference (Fig. S2, *E* and *F*). Consistent with the findings derived from Ub_4_, the D1D2 and ΔDUSP variants of both USP4 and USP15 showed robust cleavage of K48- and K63-linked Ub chains conjugated to Rsp5 (Fig. 4, *C*and *D*), reinforcing their high activity for these linkages. Thus, the distinctive regulatory role of UBL2 domain is exclusive to USP11, which is inserted between the D1 and D2 domains to direct the deubiquitinase activity of USP11 towards the K48-linked Ub chains. However, *in vitro* binding assay failed to detect the interaction of USP11 UBL2 domain alone with K29, K48, and K63-linked Ub_4_ chains and monomeric Ub (Fig. S2*G*). This suggests the necessity of UBL2 to be incorporated within the USP11 architecture for K48-chain recognition or an indirect role of UBL2 in assisting USP11 for K48-chain recognition.

**Figure 5:**
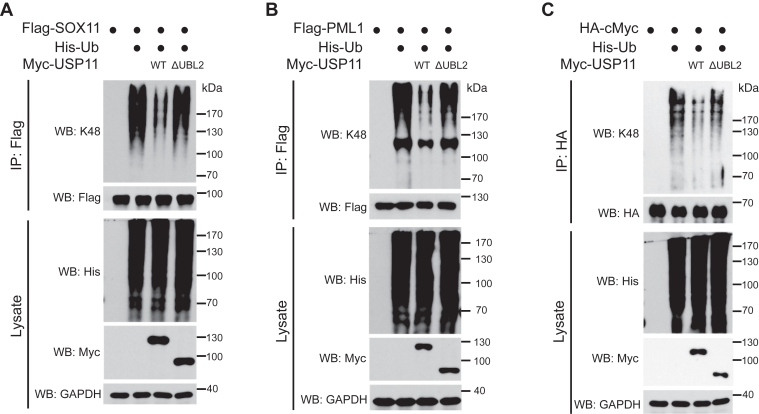
**USP11 UBL2 domain is critical for the cleavage of K48-polyubiquitinated substrates *in vivo*.***A–C*, HEK293T cells were transfected with indicated constructs and cell lysates were used for immunoprecipitation followed by Western blot analysis with indicated antibodies.

Next, we determined the impact of USP11 UBL2 domain on the removal of K48-linked chains from known USP11 substrates *in vivo*. SOX11, PML1, and c-Myc are three *bona fide* USP11 substrates *in vivo* (15, 19, 40). Although USP11 prevents their proteasomal degradation through deubiquitination, it remains undetermined for the Ub chain type that are targeted by USP11. Using a K48-chain specific antibody, we found that all three proteins were modified by this chain type in HEK293T cells (Fig. 5, *A*–*C*). Furthermore, while introducing wild type USP11 efficiently reduced K48-linked ubiquitination on SOX11, PML, and c-Myc, expression of the same amount of USP11 ΔUBL2 mutant failed to affect the K48-linked ubiquitination of these proteins (Fig. 5, *A*–*C*). These findings substantiate the critical role of UBL2 domain in guiding USP11 to recognize and cleave the K48-linked chain, in line with our *in vitro* data.

### Identification of USP11 inhibitors *via* repurposed FDA-approved pharmaceuticals

**Figure 6:**
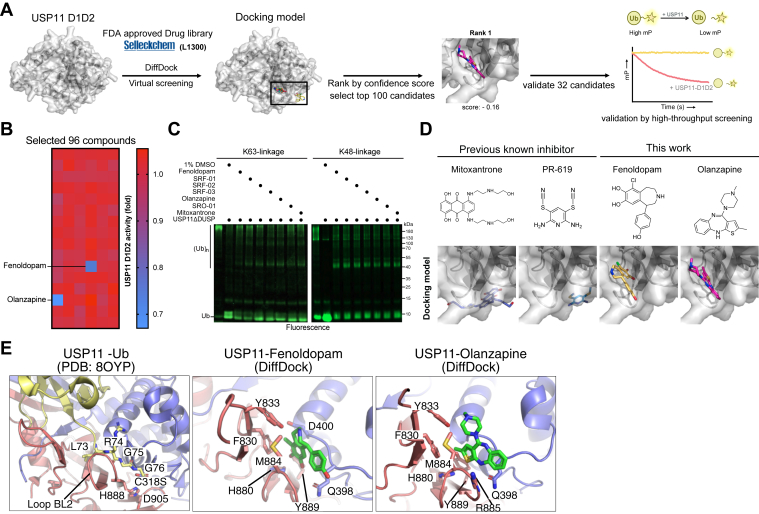
**Repurposing FDA-approved drugs to identify USP11-specific inhibitors.***A*, the structure of USP11-D1D2 was used for virtual screening of 3113 FDA-approved compounds *via* DiffDock. The *top* 100 ranked candidates were manually inspected to ensure docking at the catalytic core or S1 Ub-binding site. A total of 32 compounds were selected for *in vitro* assays. *B*, 96 compounds were tested for USP11 inhibition using Ub-FAM as a substrate. Among them, 64 compounds were originally selected from USP45 and PLpro-CoV2 screens followed by the same workflow as in (*A*) and were included as controls. Fenoldopam and Olanzapine were identified as effective inhibitors of USP11-D1D2. *C*, the indicated compounds were tested for their abilities to block the deubiquitination activity of USP11ΔDUSP2 toward K48- or K63-poyubiquitin modified substrates. *D*, chemical structures and predicted USP11–inhibitor complex models are shown. Mitoxantrone, previously reported to inhibit USP11, and PR-619, a broad-spectrum DUB inhibitor, are included for comparison. *E*, structural models of USP11 in complex with Ub, Fenoldopam, or Olanzapine suggest that the two compounds bind to a cleft overlapping with the C-terminal tail of Ub (L73–G76). Key residues involved in the interactions are labeled.

Since USP11 is associated with diseases such as cancer and neurodegeneration, the discovery of inhibitors is an emergent area to provide therapeutic potentials. Mitoxantrone, originally developed and approved as a cytotoxic anthracenedione antineoplastic agent for the treatment of acute myeloid leukemia, prostate cancer, and breast cancer, was reported to inhibit the activity of USP11 (41). With the availability of advanced tools developed by artificial intelligence (AI), we applied AI-based DiffDock (42) for virtual screening of USP11 inhibitors. DiffDock is a robust and efficient tool for structure-based virtual screening, significantly improving docking accuracy and speed compared to traditional methods. Moreover, because DiffDock does not require a predefined binding site, it allows for unbiased sampling of binding poses across the entire protein surface. We selected a commercially available, FDA-approved DUB-specific library containing 3113 compounds for the screen. To evaluate coherent selectivity, we also performed DiffDock analysis against USP45 and PLpro-CoV-2. The primary screen was conducted by evaluating the DiffDock confidence score, by which the top 100 compounds for each protease were selected. The secondary screen was performed by visual inspection to assess the existence of interactions with crucial residues around the active site of each protease and the binding poses. For each protease, 30 to 32 compounds were selected for *in vitro* activity test, thus making a total of 95 compounds for testing (Fig. 6*A*).

The *in vitro* activity test was conducted with fluorescent polarization assays using Ub-FAM as a substrate in the presence or absence of specific compounds. These assays led to the identification of Fenoldopam and Olanzapine as USP11 inhibitors. Notably, Fenoldopam and Olanzapine were selected based on DiffDock results specific to USP11, and none of the compounds selected against USP45 or PLpro showed inhibitory activity towards USP11, highlighting their specificity for USP11 (Fig. 6*B*). Fenoldopam, a selective dopamine D1 receptor agonist (43), is administered intravenously to manage severe hypertension by inducing vasodilation, thereby reducing peripheral vascular resistance and enhancing renal blood flow. Olanzapine, an atypical antipsychotic, is used to treat schizophrenia and bipolar disorder by modulating dopamine and serotonin receptors in the brain (44). To further evaluate the efficacy of these two compounds, we assessed their impacts on the deubiquitinase activity of USP11ΔDUSP using K48- and K63-linked poly-ubiquitinated Rsp5 as substrates. Similar to the broad-spectrum DUB inhibitor PR-619 (45) and a previously reported USP11 inhibitor Mitoxantrone (41), Fenoldopam and Olanzapine effectively disrupted the ability of USP11ΔDUSP to process both K48 and K63-linked Ub chains, as indicated by the retention of high-molecular-weight species in the SDS-gel (Fig. 6*C*). Furthermore, Fenoldopam and Olanzapine also inhibited the deubiquitinase activity of USP11-D1D2 using K63-linked poly-ubiquitinated Rsp5 as substrate (Fig. S3*A*). Moreover, when USP4-D1D2 and USP15-D1D2 were used for such an assay, only the broad-spectrum DUB inhibitor PR-619 showed effective inhibitory activity, while Fenoldopam and Olanzapine exhibited very weak and no detectable inhibition of USP15-D1D2 and USP4-D1D2, respectively (Fig. S3*B*), consistent with the selective nature of the AI-based DiffDock screening toward USP11.

To understand the inhibition mechanism, we analyzed the interactions of USP11-D1D2 in complex with Mitoxantrone, PR-619, Fenoldopam, and Olanzapine, utilizing the highest-ranked poses predicted by DiffDock. Mitoxantrone and PR-619 were observed to interact with the S1′ Ub site, which facilitated interactions with the Ub C-terminal tail (Fig. 6*D*), whereas Fenoldopam and Olanzapine engaged with the S1 Ub site within the catalytic cleft, which mediated Ub binding. The structural arrangement of USP11 when bound to either Fenoldopam or Olanzapine bears a striking resemblance to the inhibitor-bound configurations observed in USP7 (46) and PLpro-CoV-2 (47), showcasing a conserved mechanism of inhibition. In these instances, the inhibitors occupy the BL2 loop of catalytic core (Fig. 6*E*), effectively impeding the C-terminal tail of S1 Ub as they navigate through the catalytic cleft. Structural analysis indicated that residues Q398, F830, Y833, H880, M884, and Y889 are critical for the formation of hydrophobic interactions and hydrogen bonds with Fenoldopam and Olanzapine in proximity to the catalytic cleft.

**Figure 7:**
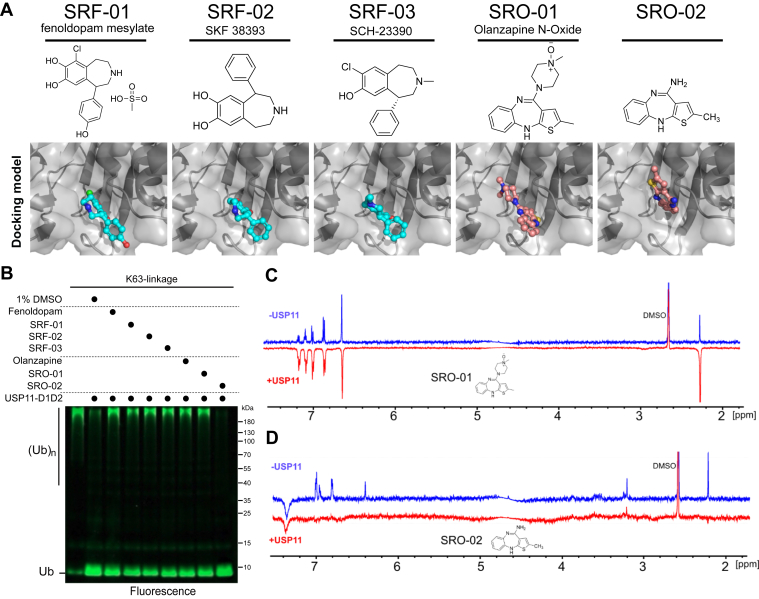
**Inhibitory validation of Fenoldopam and Olanzapine analogs.***A*, Five analogs of Fenoldopam and Olanzapine were selected for USP11 inhibition assays. Original compound names or CAS number are provided. The top-ranked DiffDock-generated structures suggested the analogs target to the same site Fenoldopam and Olanzapine target for. *B*, the indicated compounds were assayed for their activity to inhibit the deubiquitination activity of USP11-D1D2 toward K63-polyubiquitinated substrates. *C*, NMR WaterLOGSY spectra of 100 μM SRO-01 in the absence (*blue*) or presence (*red*) of 5 μM USP11-D1D2 showed opposite signal phases, while DMSO peaks remained in the same phase. These results confirm that SRO-01 binds to USP11 in solution. *D*, SRO-02 was also analyzed by WaterLOGSY, but no significant signals with opposite phase were observed, suggesting no binding or only very weak interaction below detection limits.

Next, a search for compounds sharing scaffold similarity with Fenoldopam and Olanzapine yielded five analogs, named SRF-01, SRF-02, SRF-03, SRO-1, and SRO-02, where SRF and SRO are abbreviations for scaffolds related to Fenoldopam and Olanzapine, respectively (Fig. 7*A*). Among these analogs, SRF-01, SRF-02, SRF-03, and SRO-01 showed inhibitory effects on USP11-D1-D2, while SRO-02 exhibited no inhibition (Fig. 7*B*). SRF-01, SRF-02, SRF-03, and SRO-01 also inhibited the activity of USP11ΔDUSP (Fig. 6*C*). Structural analysis comparing Olanzapine, SRO-01, and SRO-02 suggests that the piperazine ring connected to thienobenzodiazepine in Olanzapine and SRO-01 are critical for the binding of Olanzapine/SRO-01 to USP11. Specifically, in the DiffDock top-ranked structures, the piperazine group is spatially close to residues F830 and Y833 of USP11 (Fig. 6*E*), providing more interacting options between the inhibitors and the hydrophobic residues, which is essential for their inhibitory activity. NMR WaterLOGSY (48) was carried out to reveal binding event of SRO-01 and USP11-D1D2 (Fig. 7*C*), while the SRO-02 composed of thienobenzodiazepine alone did not bind to USP11-D1D2 (Fig. 7*D*), suggesting that piperazine in Olanzapine and SRO-01 is crucial for the binding with USP11-D1D2.

The inhibitory efficiency of each compound was further assessed by titrating the compound against K63-linked polyubiquitinated Rsp5 as a substrate in the reaction containing USP11-D1D2. The observed inhibitory concentrations at half maximal (IC_50_) values for Fenoldopam, Olanzapine, and their respective analogs approximated 30 μM, while those for Mitoxantrone and PR-619 were 5.8 μM and 9.5 μM, respectively (Fig. S4). These findings indicate that Fenoldopam, Olanzapine, and their analogs exhibit comparable USP11 inhibition levels to previously characterized inhibitors (Fig. S4), suggesting their potential for further development into high-potency USP11 inhibitors.

**Figure 8:**
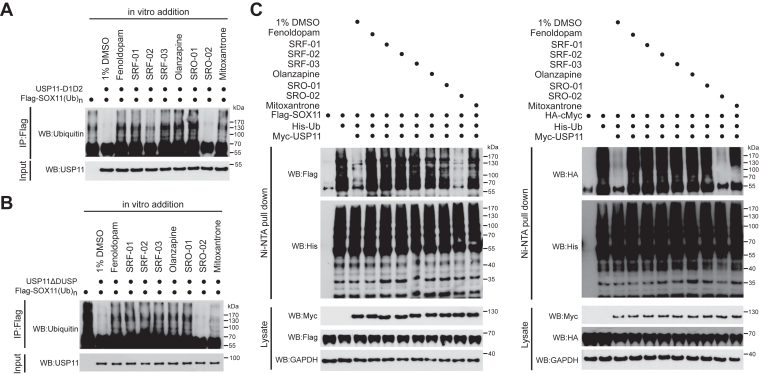
**Identified inhibitors effectively suppress USP11-mediated deubiquitination of SOX11.***A*, Ubiquitinated Flag-SOX11 was immunoprecipitated from lysates of HEK293T cells transfected with His-Ub and Flag-SOX11 and then subjected to *in vitro* deubiquitination assays with USP11ΔDUSP, together with indicated inhibitors. *B*, *C*, *in vivo* analysis of Flag-SOX11 deubiquitination in HEK293T cells transfected with His-Ub, Myc-USP11 and Flag-SOX11 (*B*) or HA-tagged c-Myc (*C*) and treated with indicated inhibitors. Among the three panels, SRO-02 was used as a negative control for showing the contrast of inhibitory efficiency by the selected inhibitors.

We further investigated whether these newly identified inhibitors affect USP11-mediated deubiquitination on SOX11, a USP11 substrate (19). USP11ΔDUSP efficiently removed the Ub chains on polyubiquitinated SOX11 isolated from transfected cells, as revealed by the elimination of high-molecular-weight bands (Fig. 8*A*). Remarkably, the six newly identified USP11 inhibitors blocked the deubiquitination activity of USP11 on SOX11, with efficacies similar to Mitoxantrone. In contrast, SRO-02 showed no inhibitory activity. This result is in agreement with those derived from *in vitro* cleavage assay, supporting that the six compounds are effective inhibitors of USP11. To inspect the *in vivo* inhibitory effects on USP11, HEK293T cells were transfected with plasmids encoding Flag-SOX11, His-Ub, and Myc-USP11, followed by treatments with the six newly identified inhibitors, Mitoxantrone, and SRO-02. *In vivo* deubiquitination assay revealed that Myc-USP11 expression remarkably decreased the poly-ubiquitination level on Flag-SOX11. Notably, the seven inhibitors significantly suppressed the deubiquitination ability of USP11, as SOX11 poly-ubiquitination levels were restored upon treatment (Fig. 8*B*). However, SRO-02 again failed to suppress USP11 activity. Using a similar approach, we found that the seven inhibitors also suppressed the activity of USP11 to deubiquitinate another substrate, c-Myc (Fig. 8*C*). These findings support that Fenoldopam, Olanzapine, and their analogs are genuine inhibitors of USP11, capable of impeding its deubiquitinase activity both *in vitro* and *in vivo*, with SOX11 and c-Myc as the substrates.

## Discussion

Our study identifies unique features of USP11 in the deubiquitination mechanism in comparison with its paralogs, USP4 and USP15, despite their high degree of homology in primary sequence and tertiary structure. First, USP11 manifests a more stringent selectivity towards different Ub chain types. More importantly, the UBL2 domain of USP11 is responsible for guiding USP11 to recognize several chain types, with K48-chains showing the most significant effect. That is, USP11 lacking UBL2 is markedly deficient in processing K48-linked chains, but UBL2 domains of USP4 and USP15 do not significantly affect chain-type recognition. These findings strongly suggest that the UBL2-IDR insert in USP11 has evolved to influence chain type recognition and highlight the essential role of non-catalytic domains in dictating DUB substrate specificity, providing new insights into the fine-tune regulation of Ub chain processing. Interestingly, USP6, USP19, and USP32 also possess an extended USP domain by incorporating a UBL domain (3). It is pertinent to investigate whether the UBLs in these three USPs assume a role in Ub chain recognition analogous to that observed in USP11.

Our finding that UBL2 domain endows USP11 with the ability to cleave K48-linked chain is in line with the results derived from many *in vivo* studies, which demonstrated the ability of USP11 to reduce the K48-polyubiquination on its cognate substrates (17, 21, 25, 26, 27, 28, 29). In addition, USP11 prevents the proteasomal degradation of many other substrates *in vivo* (15, 19, 23, 40, 49, 50, 51, 52, 53), a great portion of them likely modified by K48-linked polyubiquitin chains. Consistent with this notion, we found that several previously identified USP11 substrates, including SOX11, PML1, and c-Myc, undergo K48-polyubiquitination and that USP11 removes their Ub chains through a UBL2-dependent manner. Despite these compelling *in vivo* data, a previous *in vitro* study found that USP11 wild type and ΔUBL2 mutant manifest similar linkage selectivity and both fail to cleave K48-chains (24). The discrepancy is most likely due to the utilization of Ub_2_ chain, rather than Ub_4_ chain, in the previous study. This situation is similar to *Legionella pneumophila* deubiquitinase LotA, which displays varied deubiquitination patterns upon interacting with Ub_2_ and Ub_4_ chains (54), and underscores an advantage to use longer chains for specificity determination. This is particularly important for K48-chains, as such chains with three or more Ubs are the substrates of proteasomes (55).

**Figure 9:**
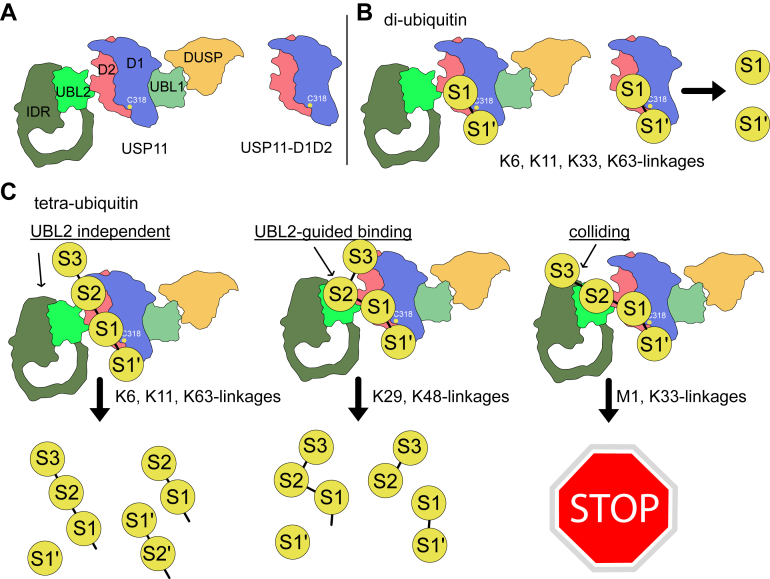
**Model of USP11-mediated polyubiquitin chain selectivity.***A*, domain organization of USP11 and USP11-D1D2 is shown with color coding consistent with Figure 1*B*. *B*, Di-Ub substrates contain a single isopeptide bond, allowing both USP11 and USP11-D1D2 to cleave K6-, K11-, K33-, and K63-linked di-Ub chains with similar specificity. *C*, in the context of tetra-Ub chains, USP11 distinguishes between linkage types through different recruitment mechanisms. K29 or K48-linked Ub_4_ is recognized *via* the UBL2 domain. In contrast, recognition of K6, K11, or K63-linked Ub_4_ does not require UBL2. This model suggests that UBL2 contributes to selective recognition and processing of K29 or K48-linked chains. M1 or K33-linkage chains may fail to bind to USP11ΔDUSP due to structural colliding to UBL2 or UBL2-IDR.

To reconcile our and previous studies, we herein propose a mechanism for the activity of USP11 to cleave K48-linked Ub chains (Fig. 9). The inability of USP11 to bind and process K48-linked Ub_2_ underscores the necessity for a longer chain to accomplish this function. A K48-linked Ub_4_ chain, being longer, allows the two distal Ub molecules to be selectively recognized by USP11 through a UBL2-guided mechanism, while the proximal Ub is processed by the catalytic core, leading to efficient deubiquitination. Since UBL2 also enhances the recognition of K29-linked Ub_4_, a similar mechanism may be applied to K29-chains. Conversely, several chain types, such as the K63-linked Ub_4_, likely adopt distinct structural orientation and thus their recognition is not dependent on UBL2. Finally, K33-linked chain is undigested by USP11ΔDUSP, implying the UBL2 domain may sterically block or substantially rearrange the interactions between the D1D2 catalytic core and K33-linked Ub_4_.

Despite the ability of UBL2 to enhance the recognition of K29- and K48-linked Ub chains, the UBL2 domain alone is incapable of binding these Ub chains. This finding suggests a requirement for UBL2 to be incorporated within the full-length USP11 architecture for binding Ub chains. Alternatively, the existence of UBL2 facilitates the exposure of a cryptic Ub-binding site on USP11. Future structural analysis is required for delineating the precise mechanism for UBL2-guided recognition of the K48-linked Ub chain.

In addition to revealing a novel recruitment and processing mechanism for K48-linked Ub chains by USP11, we identified several new inhibitors of USP11 using AI-based DiffDock screening, demonstrating a unique mechanism for deubiquitination close to the catalytic triad. These inhibitors are specific to USP11 and do not target USP4, USP15, USP45, or PLpro-CoV-2, highlighting the precision of the AI-driven approach. Although USP4, USP11, and USP15 belong to the same subfamily, the predicted binding sites of Fenoldopam and Olanzapine on USP4 and USP15 are distinct from the catalytic S1 pocket observed in USP11 (Fig. S5). Notably, Fenoldopam and Olanzapine—both FDA-approved drugs with established safety profiles—were found to inhibit USP11, suggesting a potential avenue for therapeutic intervention. We also discovered four analogs based on these two compounds, which similarly inhibit USP11, further expanding the repertoire of potential inhibitors. The fact that Fenoldopam and Olanzapine are already approved for clinical use suggests that drug repurposing may accelerate the development of USP11 inhibitors, offering a faster path to clinical application compared to the *de novo* drug development. Furthermore, the Olanzapine analogs SRO-01 and SRO-02 show distinct inhibitory efficiencies, indicating that the piperazine group is essential for their activity and underscoring the importance of specific chemical moieties in modulating the inhibitory potency. This highlights the potential of piperazine as a versatile scaffold for rational drug design and suggests that further modification of this piperazine structure could enhance USP11 inhibition, paving the way for the design of more potent and selective USP11 inhibitors.

USP11, a crucial member of the USPs family, emerges as a promising target for cancer therapy through inhibitor development. Our research exposes a previously unknown role of the UBL2 domain in determining the linkage selectivity of USP11, showcasing a unique mechanism beyond the catalytic core. Consequently, targeting the UBL2-guided mechanism in drug discovery could unlock new methods for modulating the deubiquitination of K48-linkage-specific Ub chains by USP11. In conclusion, our findings not only identify the complex regulatory mechanisms underlying USP11-mediated deubiquitination and potent micromolar-scale inhibitors of USP11 but also offer a fresh perspective on rational inhibitor design.

## Experimental procedures

### Protein expression and purification

The genes encoding USP11 variants were amplified from full-length USP11 (40) and subsequently subcloned into the pRSFDuet-1 vector (Novagen), with an N-terminal hexahistidine tag and a TEV protease cleavage site. The USP4 and USP15 variants were synthetically gene-optimized and cloned into the pRSFDuet-1 vector through a commercial service (Omics Inc). The plasmids containing these DUB genes were transformed into *E. coli* BL21 RIL cells (Agilent, USA) for overexpression. The cells were cultured in LB media until they reached an optical density of 0.6, at which point they were induced with 0.6 mM IPTG and incubated overnight at 16 °C. Ub, human UBA1, UBCH7, and the Rsp5 E3 ligase were prepared as described (56). All proteins were first purified using a gravity-based nickel affinity column (Roche), followed by TEV protease digestion to remove the N-terminal tags, and then subjected to size exclusion chromatography using Superdex 200 increase or Superdex 75 columns on an FPLC Akta (Cytiva), depending on the molecular weights of the target proteins. The pure fractions were validated by SDS-gel, concentrated, aliquoted, and flash-frozen in liquid nitrogen for long-term storage at −80 °C.

### *In vitro* DUB activity assay

The deubiquitination assays were performed using various substrates, including Ub-FAM, Ub-Rho (LifeSensors, USA), Ub_2_, Ub_4_ chains, polyubiquitinated Rsp5, and polyubiquitinated SOX11. Ub-FAM was prepared by crosslinking the additional Cys77 residue of Ub with maleimide-FAM (Santa Cruz Biotechnology). Once deubiquitination occurred, the difference of fluorescent polarization (FP) was detected to monitor the DUB activity. 2 μM Ub-FAM was incubated with 1 μM USP11 proteins in buffer containing 25 mM Tris pH 7.5, 200 mM NaCl, and 3 mM β-mercaptoethanol (βME) at room temperature for 30 min to monitor at real time for the changes of FP on a TECAN Infinite M1000 pro reader (TECAN, Switzerland).

The Ub_2_ and Ub_4_ chains were purchased from LifeSensors (LifeSensors, USA). 5 μM Ub_2_ or Ub_4_ chain was incubated with 500 nM USP11, USP4, and USP15 in buffer containing 25 mM Tris pH 7.5, 200 mM NaCl, and 3 mM βME at room temperature and quenched by 2X SDS sample buffer at indicated time points. The deubiquitination results were validated by Western blot using anti-Ub antibody.

Polyubiquitinated Rsp5 was made in-house by mixing 1 μM human UBA1, 1 μM UBCH7, 1 μM Rsp5 E3 ligase (residues 383–809, K63-linkage) or a Rsp5 Q808M-E809L mutant form (K48 linkage), and 10 μM fluorescein-labeled Ub with the supplement of 20 mM MgCl_2_, and 20 mM ATP in buffer containing 25 mM HEPES pH 7.0, and 200 mM NaCl. The reaction was carried out at 37 °C for 3 h and quenched by 100 mM EDTA. DUB activity was measured by adding 1 μM of USP proteins and monitoring the decreased length of poly-ubiquitinated chains in the SDS-gel at designated experimental times.

Polyubiquitinated Flag-SOX11 was isolated from HEK293T cells transfected with His-Ub and Flag-SOX11 using immunoprecipitation with anti-Flag beads (Thermo Fisher). The bead-bound proteins were then incubated with 5 μM USP11 D1D2 or USP11 ΔDUSP and treated with the following compounds: 0.5 μM mitoxantrone, 3 μM Fenoldopam, 3 μM SRF-01, 3 μM SRF-02, 3 μM SRF-03, 3 μM Olanzapine, or 3 μM SRO-01. Reactions were carried out at 37 °C for 2 h in a 40 μl mixture containing 20 mM Tris-HCl (pH 7.6), 150 mM NaCl, 0.1% Triton X-100, 0.2% NP-40, and 10 mM DTT. After incubation, the beads were washed 3 times with TBST buffer (20 mM Tris-HCl pH 7.5, 150 mM NaCl, and 0.1% Tween-20) and then analyzed by Western blot.

### *In vitro* binding

2 μM His-USP11 UBL2 proteins and 2 μM K29, K48, and K63-linked Ub_4_ were mixed and incubated at 37 °C for 1 h to allow potential binding. Following incubation, the Ni-NTA agarose (Cytiva) was added and incubated at 4 °C for 2 h. The beads were washed three times with PBS, and the bound proteins were analyzed by Western blot.

### Western blot analysis

The cells were lysed by RIPA lysis buffer containing 150 mM NaCl, 20 mM Tris-HCl pH 7.5, 1% NP40, 0.1% SDS, 1% sodium deoxycholate, 1 μg/ml aprotinin, 1 μg/ml leupeptin, and 1 mM phenylmethylsulphonyl fluoride (PMSF). Total protein was measured using a Bradford reagent. For sample preparation, protein extracts with sample buffer (50 mM Tris pH 6.8, 2% SDS, 2.5% βME, 10% glycerol, and 0.02% bromophenol blue) were incubated at 95 °C for 10 min. Proteins were separated by SDS-PAGE, then transferred on polyvinylidene difluoride (PVDF) membrane. The PVDF membrane was stained by primary antibodies at 4 °C overnight and secondary antibodies (GE Healthcare) for 1 h at room temperature. Membranes were probed with Immobilon Western Chemiluminescent HRP Substrate (PerkinElmer Inc). The antibodies used are listed in Table S1.

### Cell culture, transfection, and plasmids

HEK293T cell lines were obtained from the American Type Culture Collection (ATCC, Manassas, VA, USA). Cells were cultured in Dulbecco's modified Eagle medium (DMEM) supplemented with 10% fetal bovine serum (FBS) and 1% penicillin-streptomycin (P/S) incubated at 37 °C with 5% CO_2_ in the air. Transfection was performed with the calcium phosphate method. The constructs for His-Ub, Myc-USP11, Flag-SOX11, and Flag-PML1 were described previously (19, 40), whereas HA-c-Myc was obtained from Yu-Ru Lee (Academia Sinica, Taiwan). Myc-USP11 ΔUBL2 was generated by site-directed mutagenesis using primers designed to amplify two overlapping fragments flanking the UBL2 domain. Following amplification, the reaction mixture was treated with DpnI (NEB) at 37 °C for 1 h to digest the parental methylated plasmid.

### *In vivo* deubiquitination

Cells transfected with Flag-SOX11 or HA-tagged c-Myc along with His-Ub and Myc-USP11 were treated with 0.5 μM mitoxantrone, 3 μM fenoldopam, 3 μM SRF-01, 3 μM SRF-02, 3 μM SRF-03, 3 μM olanzapine, 3 μM SRO-02, or 3 μM SRO-01 for 16 h, followed by treatment with 5 μM MG132 (Calbiochem) for 6 h. Cells were lysed using buffer A (6 M guanidine-hydrochloride, 0.1 M Na_2_HPO_4_/NaH_2_PO_4,_ pH 8.0 and 10 mM imidazole), and the lysates were incubated with Ni-NTA agarose (Cytiva) for 2 h at 4 °C. The beads were then washed twice with a mixture of buffer A and buffer TI (25 mM Tris-HCl, pH 6.8 and 20 mM imidazole) at a volume ratio of 1:3, followed by five times with buffer TI and then analyzed by Western blot.

For assaying K48-linked ubiquitination, cells transfected with His-Ub, Myc-USP11 WT or mutant, together with Flag- or HA-tagged substrates, were treated as described above. Cells were lysed with immunoprecipitation lysis buffer containing 50 mM Tris (pH 8.0), 0.15 M NaCl, 1% NP-40, 1% sodium deoxycholate, 0.1% SDS, 1 mM PMSF, aprotinin (1 μg/ml), and leupeptin (1 μg/ml), and then lysates were incubated with anti-Flag beads (Thermo Fisher) or anti-HA beads (Thermo Fisher) for 1 h at 4 °C. The beads were washed with TBST buffer, followed by Western blot analysis with anti-K48 Ub antibody.

### Discovery of potential inhibitors

To discover small-molecule inhibitors for USP11, we employed DiffDock to simulate the binding poses of various compounds to the catalytic domain of USP11. A total of 3113 FDA-approved ligands commercially available from Selleckhem (catalog number: L1300) were used to simulate the USP11-ligand complex structures. The AlphaFold structure of the USP11 D1-D2 region was used as the template for docking. The top 100 highest-scoring chemicals were further inspected to ensure the ligands reside in or near the catalytic pocket, blocking USP11-Ub interactions. In addition to USP11, USP45 and PLpro-CoV-2 were also included in the search for specific inhibitors. 32 selected chemicals for each DUB were ordered, and their inhibitory activities were validated using *in vitro* DUB assays with Ub-FAM as the substrate. Fenoldopam and olanzapine, identified from the USP11 screen, were found to specifically inhibit USP11 activity. The two compounds were also used for DiffDock prediction for USP4-D1D2 (PDB ID 2Y6E) and USP15-D1D2 (PDB ID 6GHA). Based on the chemical scaffolds, we further searched the PubChem chemical database and identified several analogs for DiffDock to expand the inhibitor library. Five more candidates, SRF-01, SRF-02, SRF-03, SRO-01, and SRO-02, were selected for further *in vitro* efficiency validation. SMILES information for the listed compounds are summarized in Table S2.

### NMR spectroscopy

To assess the interaction between the protein and the ligand, WaterLOGSY (48) experiments were performed, adhering to a previously established protocol (57), to characterize the binding events between USP11-D1D2 and individual compounds. Briefly, compounds were dissolved in deuterated DMSO (Cambrdige Isotope Laboratories) and subsequently mixed with USP11-D1D2 in PBS buffer, resulting in final concentrations of 5 μM and 100 μM for the protein and compounds, respectively. Additional NMR samples, excluding the protein, were utilized as controls. The NMR WaterLOGSY experiments were conducted at 25 °C using a Bruker HD AVANCE III 600 MHz spectrometer equipped with a BBFO probe. Each 1D WaterLOGSY spectrum was acquired with a mixing time of 2 s and a relaxation delay of 2 s, and was referenced to trimethylsilylpropanoic acid (TSP) at 0 ppm. The spectra were then processed using Topspin 4.3 (Bruker BioSpin, Germany).

### Enzymatic kinetics

Ub-Rho was used to measure the USP11 catalytic efficiency *k*_*ca*t_/K_M_. 50 to 100 nM USP11 proteins and 1 μM substrate were mixed in buffer containing 25 mM Tris pH 7.5, 200 mM NaCl, and 3 mM βME to detect the increased fluorescent signals when Rhodamine drops off from the attached Ub (35).

The IC_50_ values of selected inhibitors were measured by mixing 1 μM USP11-D1D2 and poly-ubiquitinated Rsp5 with various concentrations of inhibitors in buffer containing 25 mM Tris pH 7.5, 200 mM NaCl, and 3 mM βME to observe the efficiency of deubiquitination in SDS-gel. The accumulated monomeric Ub fluorescent signals were integrated and plotted as a function of inhibitor concentration using GraphPad Prism to calculate the IC_50_ values. The fitting equation is provided below.

$$y=100\;/\left[1+{\left(\frac{x}{IC50}\right)}^{Hill\;slope}\right]$$

The Y values were normalized between 100% and 0% for the fitting, where “Hill slope” was also included for fitting and the values were always around 1.0.

## Data availability

All data are presented in the main figures and online Supplementary Information. Raw data and computational files are available upon request.

## Supporting information

This article contains supporting information.

## Conflict of interest

The authors declare that they have no conflicts of interest with the contents of this article.
