# Supplementary material for: Non-catalytic UBL2 domain directs deubiquitinase USP11 toward K48-linked polyubiquitin chains
Source: J Biol Chem. 2025 Nov 7;301(12):110924. doi: 10.1016/j.jbc.2025.110924 (PMC12718135; doi:10.1016/j.jbc.2025.110924)
Supplement: Supporting Figures and Tables [file mmc1.docx]

**Supplementary Information**

**Non-catalytic UBL2 domain directs deubiquitinase USP11 toward K48-linked polyubiquitin chains**

Sin-Rong Lee^1,2^, Han-Hsiun Chen^1,2^, Ruey-Hwa Chen^1,2,*^, and Kuen-Phon Wu^1,2,*^

1. Institute of Biological Chemistry, Academia Sinica, Taipei, Taiwan

2. Institute of Biochemical Sciences, College of Life Science, National Taiwan University, Taipei, Taiwan

*: correspondence:

Ruey-Hwa Chen: [rhchen@as.edu.tw](mailto:rhchen@as.edu.tw)

Kuen-Phon Wu: [kpwu@as.edu.tw](mailto:kpwu@as.edu.tw)


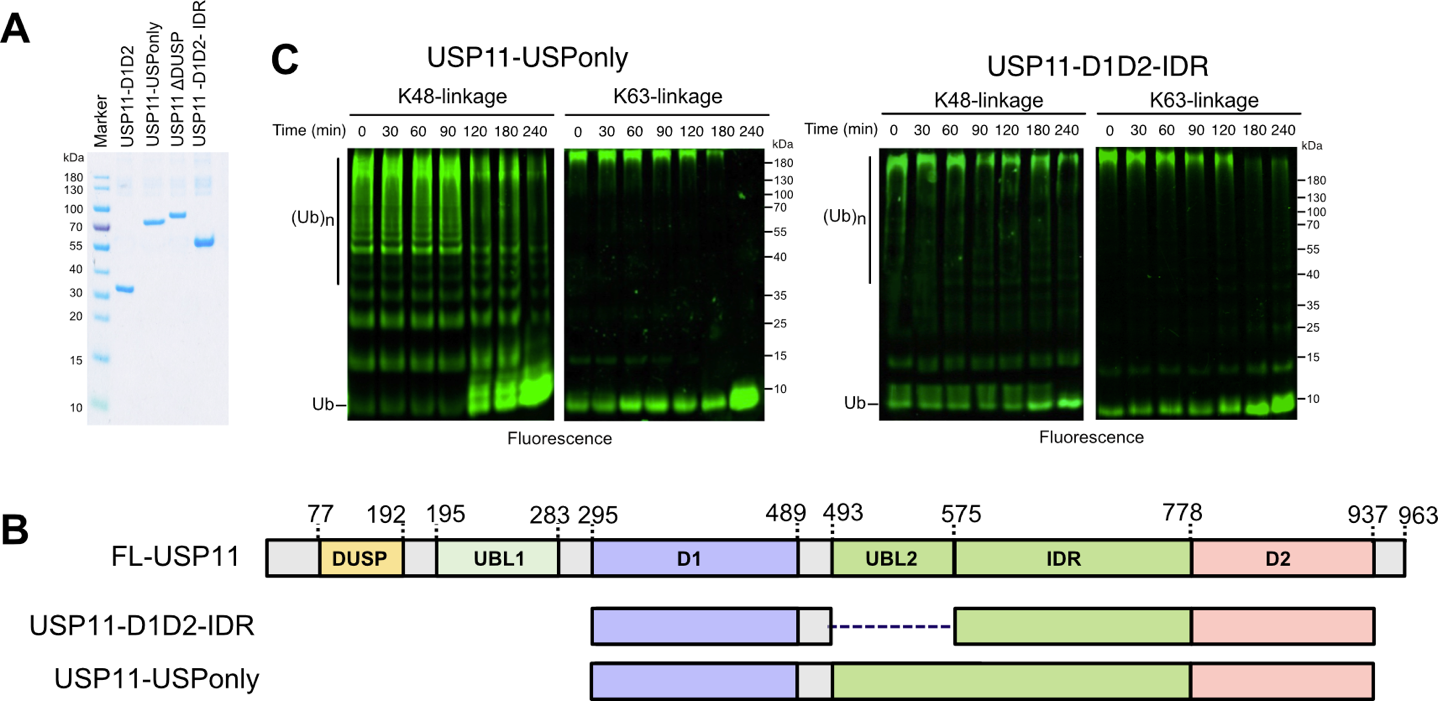


**Figure S1. Additional USP11 variants support the role of UBL2 in recognizing K48-linked Ub chains.**

1. The purity of all USP11 variants generated by this study

**(B)** Domain architectures of two additional USP11 variants USP11-D1D2-IDR and USP11-USPonly.
**(C)** USP11-USPonly, but not USP11-D1D2-IDR, efficiently cleaved K48-linked polyubiquitinated Rsp5 substrates, indicating that UBL2 is involved in recruiting K48-linked chains for processing.


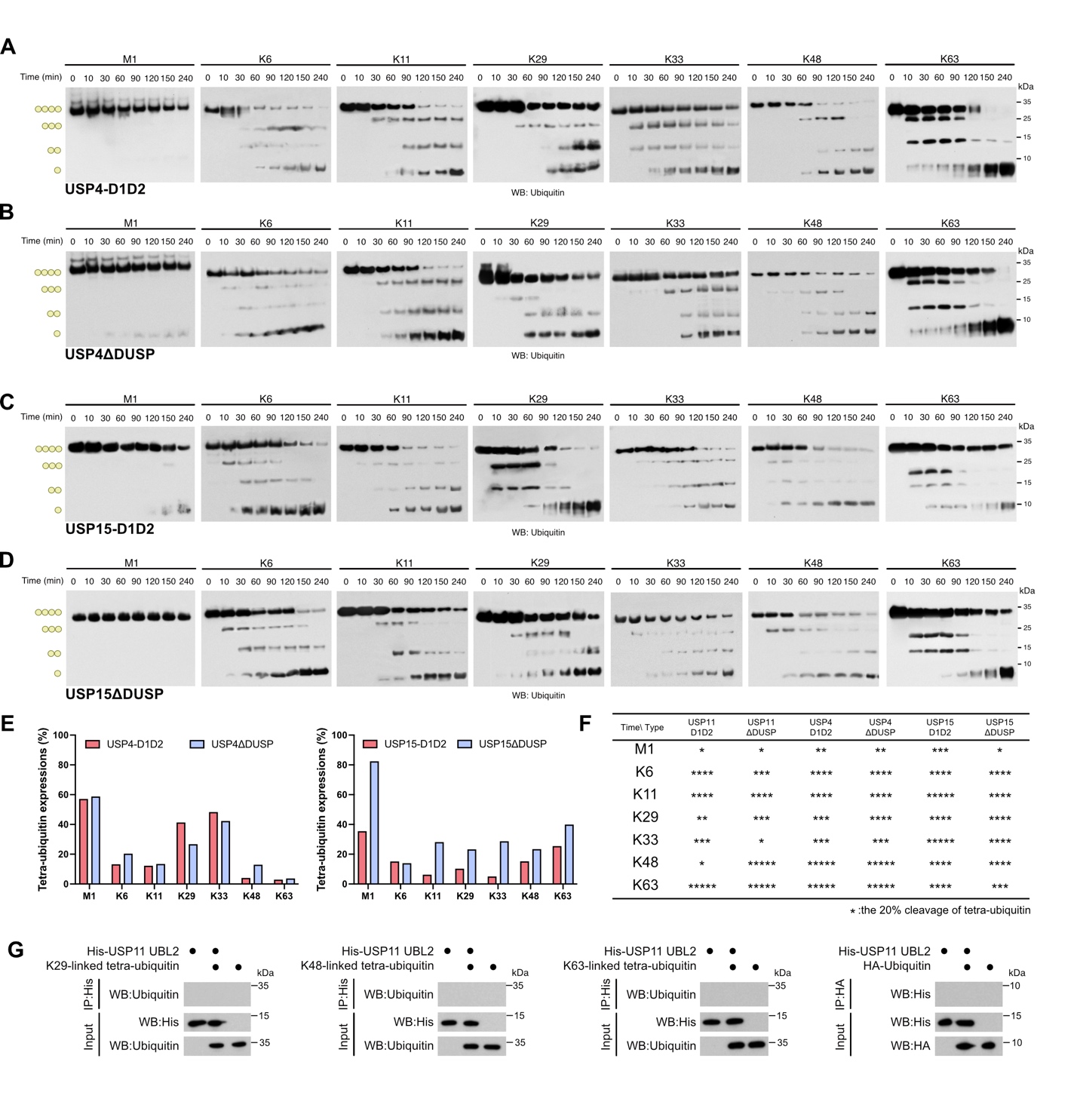


**Figure S2. Ubiquitin chain selectivity of USP4 and USP15 variants.**

**(A, B)** Ub_4_ chains were used to examine domain-specific selectivity of USP4-D1D2 and USP4∆DUSP.
**(C, D)** USP15-D1D2 and USP15∆DUSP were tested for Ub_4_ chain selectivity. No major differences were observed, except that M1-linked Ub_4_ chains were more efficiently cleaved by USP15-D1D2.
**(E)** Quantification of the residual Ub_4_ chains from panels A–D shows similar digestion patterns between each variant pair.
**(F)** Cleavage efficiencies for the seven Ub_4_ linkages by the four variants are summarized and ranked using one to five asterisks where one asterisk stands for reduction of 20% Ub_4_ chains. A greater number of asterisks indicates higher deubiquitination efficiency.

**(G)** UBL2 domain alone was purified to test its binding to Ub monomer or indicated Ub chains. No interaction was detected.


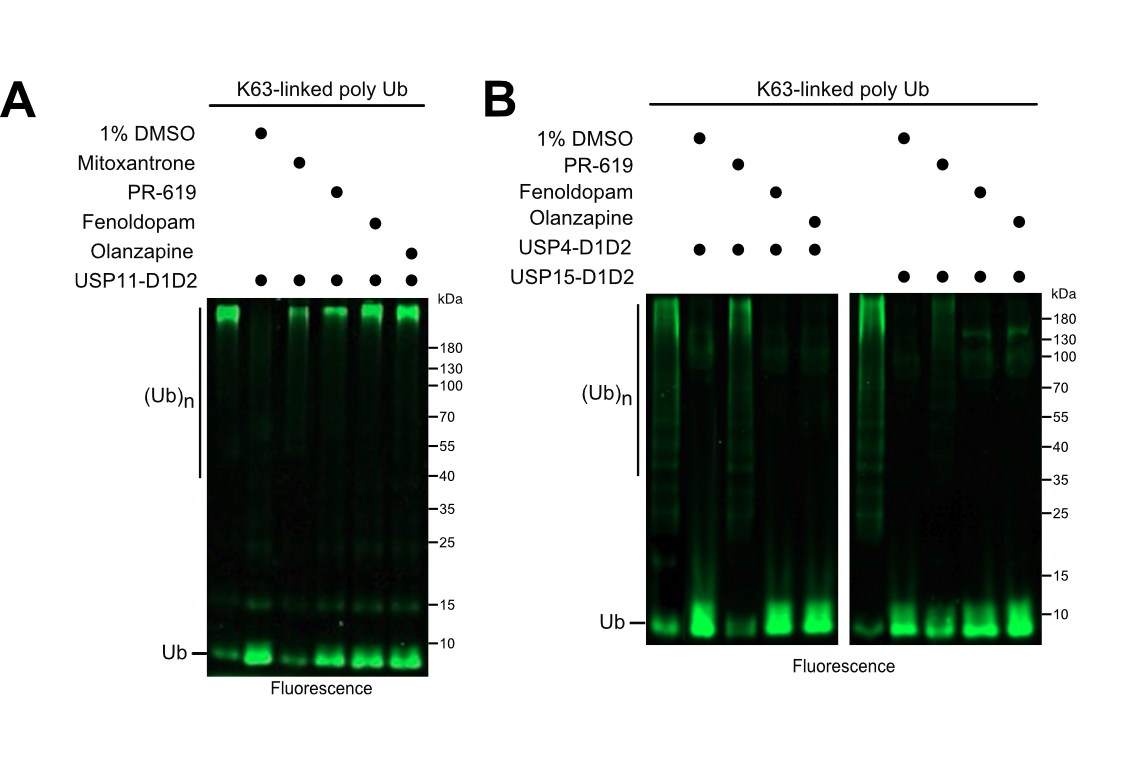


**Figure S3. The effect of identified inhibitors on the activity of USP11-D1D2, USP4-D1D2, and USP15-D1D2.**

**(A)** The four compounds were tested with USP11-D1D2 using polyubiquitin substrates. Partial retention of K63-linked chains was observed upon treatment, indicating effective inhibition.

**(B)** The four compounds were also tested against USP4 and USP15. Only PR-619 inhibited both, while Fenoldopam, Olanzapine, and Mitoxantrone showed no inhibitory effect, suggesting USP11 specificity.


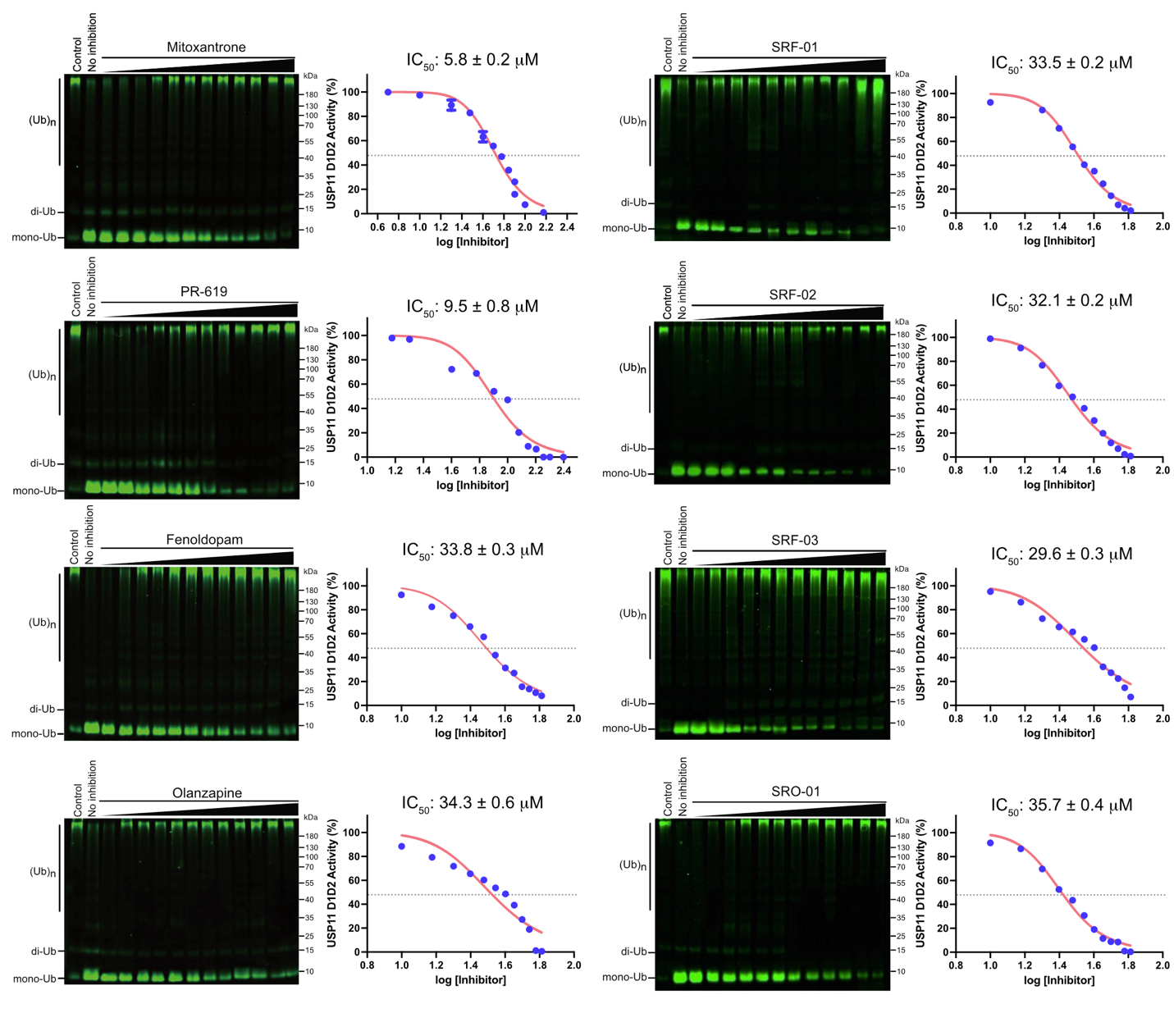


**Figure S4. Estimation of IC₅₀ values for selected USP11 inhibitors.**

4 µM USP11-D1D2 was incubated with 5 µM polyubiquitinated Rsp5 in the presence of varying concentrations of inhibitors. Reactions were performed at 37 °C for 3 hours and analyzed by fluorescein detection after electrophoresis. USP11 activity was estimated based on the remaining high-molecular-weight Rsp5-Ubₙ species, and IC₅₀ values were calculated accordingly.


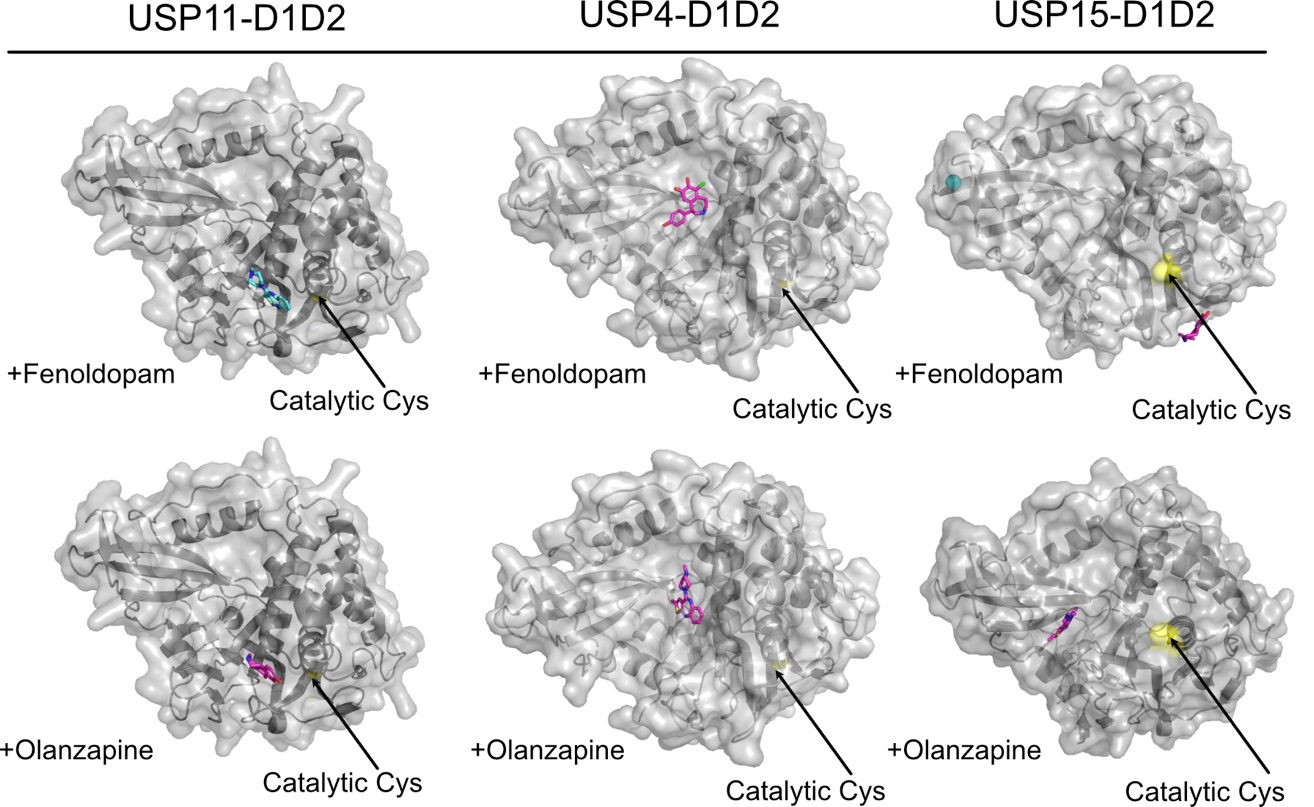


**Figure S5. Complex structures of Fenoldpam and Olanzapine with the D1D2 versions of USP4, USP11, and USP15 predicted by DiffDock.**

The USP4-D1D2 (PDB ID 2Y6E) and USP15-D1D2 (PDB ID 6GHA) crystal structures were used to dock with Fenoldpam and Olanzapine. Top ranked configuration of the two compounds docked in USP4 and USP15 reveal distinct binding site from the USP11-D1D2 results. Catalytic cysteines of the three USP proteins are colored in yellow and indicated.

**Table S1: Antibodies used in this study**

| **Antibody** | **Vendor** | **Catlog number** | **Source** | **Titer** |
| --- | --- | --- | --- | --- |
| 6xHis | Takara Bio | 631212 | Mouse | WB(1:5000) |
| Flag | GeneTex | GTX115043 | Rabbit | WB(1:1000) |
| HA | Cell Signaling | 3724 | Rabbit | WB(1:1000) |
| Myc | Cell Signaling | 2278 | Rabbit | WB(1:1000) |
| GAPDH | GeneTex | GTX100118 | Rabbit | WB(1:1000) |
| Ubiquitin | Cell Signaling | 3936 | Mouse | WB(1:1000) |
| K48-linked Ub | Cell Signaling | 8081 | Rabbit | WB(1:1000) |
| K63-linked Ub | Cell Signaling | 5621 | Rabbit | WB(1:1000) |
| USP11 | Abcam | ab191235 | Mouse | WB(1:1000) |
| mouse IgG (HRP) | GE Healthcare | NA931-1ML | N/A | WB(1:5000) |
| rabbit IgG (HRP) | GE Healthcare | NA934-1ML | N/A | WB(1:5000) |
|  |  |  |  |  |

**Table S2: SMILES information of the chemical compounds used this study**

| ID / Name | Canonical SMILES |
| --- | --- |
| Fenoldopam | OC1=CC=C(C=C1)C2CNCCC3=C(Cl)C(=C(O)C=C23)O |
| Olanzapine | CN1CCN(CC1)C2=NC3=C(NC4=C2C=C(C)S4)C=CC=C3 |
| Mitoxantrone | C1=C(C(=NC(=C1SC#N)N)N)SC#N |
| PR-619 | C1=CC(=C2C(=C1NCCNCCO)C(=O)C3=C(C=CC(=C3C2=O)O)O)NCCNCCO |
| SRF-01 | CS(=O)(=O)O.C1CNCC(C2=CC(=C(C(=C21)Cl)O)O)C3=CC=C(C=C3)O |
| SRF-02 | OC1=C(O)C=C2C(C3=CC=CC=C3)CNCCC2=C1.[H]Cl |
| SRF-03 | OC1=C(Cl)C=C2CCN(C)C[C@H](C3=CC=CC=C3)C2=C1.[H]Cl |
| SRO-01 | CC1=CC2=C(S1)NC3=CC=CC=C3N=C2N.Cl |
| SRO-02 | CC1=CC2=C(NC3=CC=CC=C3N=C2N4CC[N+]([O-])(CC4)C)S1 |
